# Reconstruction of massive bone defects after femoral tumor resection using two new-designed 3D-printed intercalary prostheses: a clinical analytic study with the cooperative utilization of multiple technologies

**DOI:** 10.1186/s12891-023-06171-w

**Published:** 2023-01-25

**Authors:** Xianhao Shao, Mengmeng Dou, Qiang Yang, Jianmin Li, Ailin Zhang, Yuan Yao, Qing Chu, Ka Li, Zhenfeng Li

**Affiliations:** 1 Department of Orthopaedics, Shandong Provincial Hospital Affiliated to Shandong First Medical University, Jinan, 250021 Shandong China; 2 Department of Orthopaedics, Qilu Hospital, Cheeloo College of Medicine, Shandong University, Jinan, 250012 China; 3 Department of Biomedical Engineering, Tianjin First Central Hospital, Tianjin, 300070 China; 4 Physiotherapy department, Acute Neurosciences, the Wesley Hospital, 451 Coronation Drive, Auchenflower, QLD 4066 Australia; 5 Department of Radiography, Qilu Hospital, Cheeloo College of Medicine, Shandong University, Jinan, 250012 China; 6 State Key Laboratory of Cardiovascular Disease, Fuwai Hospital, National Centre for Cardiovascular Diseases, Chinese Academy of Medical Sciences and Peking Union Medical College, Beijing, 100037 China; 7 Department of Cardiac Surgery, Fuwai Hospital, Chinese Academy of Medical Science and Peking Union Medical College, Beijing, 100037 China

**Keywords:** 3D printing, Intercalary prosthesis, Femur, Bone tumor resection, Reconstruction, Joint-preserving surgery

## Abstract

**Background:**

To reconstruct massive bone defects of the femoral diaphysis and proximal end with limited bilateral cortical bone after joint-preserving musculoskeletal tumor resections, two novel 3D-printed customized intercalary femoral prostheses were applied.

**Methods:**

A series of nine patients with malignancies who received these novel 3D-printed prostheses were retrospectively studied between July 2018 and November 2021. The proximal and diaphyseal femur was divided into three regions of interest (ROIs) according to anatomic landmarks, and anatomic measurements were conducted on 50 computed tomography images showing normal femurs. Based on the individual implant-involved ROIs, osteotomy level, and anatomical and biomechanical features, two alternative 3D-printed prostheses were designed. In each patient, Hounsfield Unit (HU) value thresholding and finite element analysis were conducted to identify the bone trabecula and calcar femorale and to determine the stress distribution, respectively. We described the characteristics of each prosthesis and surgical procedure and recorded the intraoperative data. All patients underwent regular postoperative follow-up, in which the clinical, functional and radiographical outcomes were evaluated.

**Results:**

With the ROI division and radiographic measurements, insufficient bilateral cortical bones for anchoring the traditional stem were verified in the normal proximal femur. Therefore, two 3D-printed intercalary endoprostheses, a Type A prosthesis with a proximal curved stem and a Type B prosthesis with a proximal anchorage-slot and corresponding locking screws, were designed. Based on HU value thresholding and finite element analysis, the 3D-printed proximal stems in all prostheses maximally preserved the trabecular bone and calcar femorale and optimized the biomechanical distribution, as did the proximal screws. With the 3D-printed osteotomy guide plates and reaming guide plates, all patients underwent the operation uneventfully with a satisfactory duration (325.00 ± 62.60 min) and bleeding volume (922.22 ± 222.36 ml). In the follow-up, Harris Hip and Musculoskeletal Tumor Society scores were ameliorated after surgery (*P* < 0.001 and *P* < 0.001, respectively), reliable bone ingrowth was observed, and no major complications occurred.

**Conclusions:**

Two novel 3D-printed femoral intercalary prostheses, which achieved acceptable overall postoperative outcomes, were used as appropriate alternatives for oncologic patients with massive bone defects and limited residual bone and increased the opportunities for joint‐preserving tumor resection. Several scientific methodologies utilized in this study may promote the clinical design proposals of 3D-printed implants.

## Background

The femur is regarded as a vital biomechanical load-bearing structure in the musculoskeletal system, in which its proximal end strongly contributes to not only load-transfer, but also load-carrying [1, 2]. Based on the development of surgical techniques and implant materials, considerable scientific investigations have been carried out to improve oncological segmental resection, which requires the reconstruction of intercalary prostheses in the extremities [3]. However, how to preserve more anatomical structures and how to acquire better postoperative prognosis while ensuring safe surgical margins remain difficult issues when applying an intercalary prosthesis that involves both diaphyseal and proximal metaphyseal regions to reconstitute segmental massive bone defects, particularly in the femur [4, 5]. Generally, there exists a pivotal point that the guarantee of steady bone-implant integration and acceptable postoperative function often requires sufficiently long and thick stems in modular or customized prostheses, which are utilized in the segmental resection of diaphyseal tumors [6], and Ahlmann et al. proposed that the implant stem should be applied at least 5.0 cm to stabilize the internal fixation [7]. However, if the bilateral residual cortical bones are too short to anchor the stem firmly, unreliable biomechanical stability may appear even though auxiliary screws and plates are utilized [8]. Therefore, it is easy to cause the poor functional prognosis and micromotion of implants when choosing the traditional segmental prosthesis to reconstruct a massive bone defect of the proximal and diaphyseal region in the femur with inadequate bilateral cortical bone fixation [9]. However, if surgery that sacrifices the articular facet is undertaken to improve the stability of fixation, the occurrence rate of long-term implant-related adverse events and prosthetic revision may increase [10]. Unlike the diaphysis, which possesses bilateral cortical bone, the trabecular bone, one of the cardinal components in the cancellous bone of the proximal femur, plays an important role in load transfer and energy absorption [1, 2]. In addition, the calcar femorale, which is a dense internal septum reaching from the femoral neck to the distal lesser trochanter, is critical in dealing with the complex forces that occur in the proximal femur [11, 12]. Theoretically, a more efficient utilization of anatomic structure and biomechanics may be realized if sophisticated consideration is given to the coordination between calcar femorale, trabecular bone, and implant when the customized prostheses are designed, thereby improving postoperative radiographic and functional outcomes. Based on above clinical background, the design of a novel femoral intercalary prosthesis, which may promote the overall prognosis after reconstruction for oncologic massive bone defects that involve the femoral diaphysis and proximal end and provide a more precise and steady combination of the implant and residual bone, urgently warrants further research to benefit relevant patients.

Three-dimensional printing technology has enormously promoted the development of basic orthopedic investigations in the past decade [13, 14]; however, relatively few studies based on this emerging technology have been conducted in clinical research, especially in the realm of bone oncology, in which major investigations focus on 3D-printed implants for the reconstruction of spines, pelvises and large diarthroses [15–17]. In contrast, limited investigations have been carried out for the application of 3D-printed intercalary prostheses to reconstruct the diaphyseal and metaphyseal regions caused by massive bone defects in the extremities [18–20]. Zhao et al. described the application of 3D‐printed intercalary prostheses for tibial “ultracritical sized bone”, which achieved satisfactory overall early biological fixation and limb function [18]; however, femoral 3D‐printed intercalary prostheses are rarely elaborated and evaluated [19]. To the best of our knowledge, the new-designed 3D-printed femoral intercalary prostheses applied to the reconstruction of oncological massive bone defects that involved the femoral diaphysis and proximal metaphysis were never introduced in the previous scientific article, nor were the design concept, region division, postoperative functional assessment or anatomic analysis elucidated for such implants.

We conducted this investigation mainly for the following reasons: (1) The individual 3D-printed design may maximally reserve the anatomic structures and rationalize the stress distribution in the proximal femur, which participates in load transfer and load carrying. (2) Novel 3D-printed stems may enable the preservation of the femoral proximal articular facet in patients with a limited length of residual proximal bilateral cortical bones. (3) With the 3D customized bone-implant interfaces that facilitate bone ingrowth and osseointegration and the new-designed endoprostheses that take into account both mechanical and biological reconstruction, the postoperative function in such patients may be improved. In addition, the techniques based on the Hounsfield Unit (HU) value can distinguish calcar femorale and trabecular bone from their peripheral intraosseous spaces, and finite element analysis (FEA) can be manipulated to finely analyze the biomechanics distribution in different regions. However, these technologies have never been clinically implemented and elaborated in the design of tumor-related 3D-printed femoral intercalary prostheses to the best of our understanding. Based on the background above, this study aims to systemically investigate the clinical application and overall prognosis of two types of novel 3D-printed femoral intercalary prostheses, which were designed with the comprehensive consideration of multiple factors, such as region of interest (ROI) division, radiographic measurement, anatomic identification, and finite element mechanical analysis.

## Materials and methods

### Patient characteristics

This study was conducted in Shandong Provincial Hospital Affiliated to Shandong First Medical University and Qilu Hospital of Shandong University with the approval of the institutional ethics committee. Whether a patient was eligible was judged by the inclusion criteria and exclusion criteria below.

#### Inclusion criteria

1. Malignant or aggressive tumors with definite diagnosis. 2. Resectable Lesions [21]. 3. Lesions that are more suitable for segmental resection rather than curettage, radiofrequency ablation, or other surgical/nonsurgical treatments. 4. The resected segment involves the diaphyseal and proximal femur but does not reach the proximal epiphysis. 5. The bone defect is appropriately reconstructed by 3D-printed intercalary prostheses.

#### Exclusion criteria

 1. The diaphyseal and proximal lesion reaches the degree for total hip arthroplasty or extra-articular resection. 2. Patients with open physes. 3. Patients who underwent previous operations that may affect the functional prognosis. 4. Patients with surgical contraindications. 5. Patients who are ineligible for the preplanned surgery due to poor responses to chemotherapy. 6. Patients who did not consent to the inclusion of their private clinical information in this study.

Nine patients (eight males and one females), with an average age of 42.7 ± 22.4 years (18–69 years old), who underwent the reconstruction of 3D-printed intercalary endoprostheses for the femoral diaphyseal and proximal oncological bone defect in our hospital were enrolled in this clinical study from July 2018 to November 2021, with the specific patient demographics displayed anonymously (Table 1).

**Table 1:** Patient demographics

ID/S/A	Site	Pathological diagnosis	Preoperative therapy	Postoperative therapy
1/M/30	L	Rhabdomyosarcoma	CH	CH
2/F/18	R	Osteosarcoma	CH	CH
3/M/56	R	Osteosarcoma	CH	CH
4/M/69	R	Chondrosarcoma	-	-
5/M/69	R	Ewing sarcoma	-	CH
6/M/19	R	Osteosarcoma	CH	CH
7/M/67	L	Chondrosarcoma	-	-
8/M/35	R	Metastatic tumor (laryngeal cancer)	TR for the primary site + CH	CH
9/M/21	L	Osteosarcoma	CH	CH

*CH* chemotherapy, *TR* tumor resection

### Defining the femoral diaphyseal and proximal metaphyseal regions

**Fig. 1:**
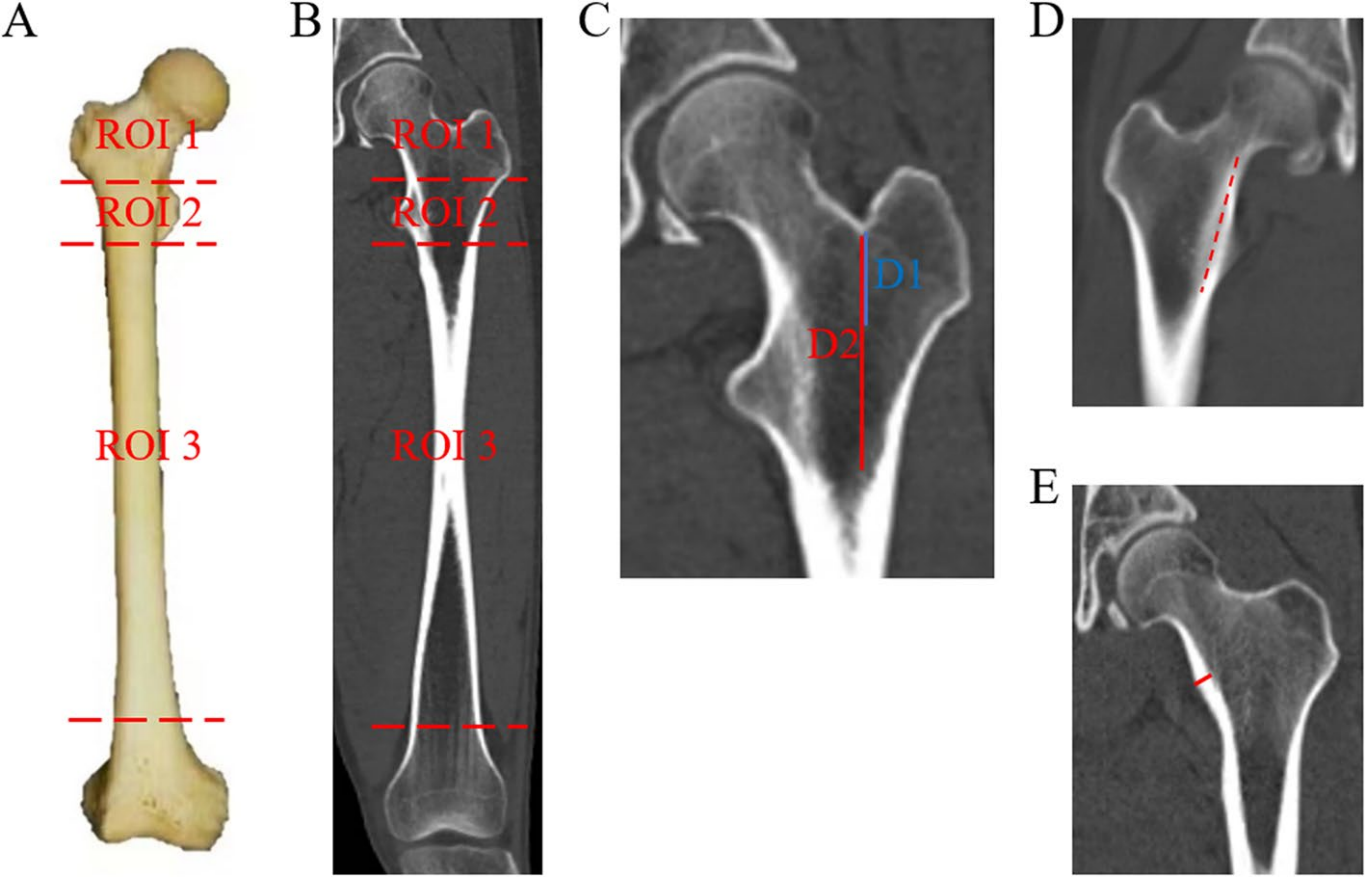
The division of the region of interest (ROI) and relevant radiographic measurements. **A** Graphical illustration of ROI-1, ROI-2 and ROI-3. **B** The radiographic identification of ROI-1, ROI-2 and ROI-3. **C** The measurements of the distance between the trochanteric fossa and the superior edge of the lesser trochanter (D1) expressed as a blue line, and the distance between the trochanteric fossa and the inferior edge of the lesser trochanter (D2) expressed as a red line. **D** Measurement of the calcar femorale (red dashed line). **E** Measurement of calcar femorale thickness (red line)

The reconstructed sagittal and coronal computed tomography (CT) images (showed femur) from 50 normal adults were used for radiographic measurement and analysis by dividing the proximal and diaphyseal femur into three ROIs. Based on the anatomic landmarks, ROI-1, ROI-2 and ROI-3 were referred to as the region superior to the superior edge of the lesser trochanter (femoral head-neck region), the region between the superior and inferior edges of the lesser trochanter (femoral intertrochanteric region), and the region between the inferior edge of the lesser trochanter and femoral distal metaphysis (femoral diaphyseal region), respectively (Fig. 1A, B). The measured parameters included the distance between the trochanteric fossa and the superior edge of the lesser trochanter (D1) and the distance between the trochanteric fossa and the inferior edge of the lesser trochanter (D2) (Fig. 1C). We recorded the maximum valid length of bilateral cortical bones in ROI-1 and ROI-2 according to the previous criteria [22, 23]. The calcar femorale and its thickness and density were measured as previously described [11, 24, 25] (Fig. 1D, E).

### Design and properties of prostheses

**Fig. 2:**
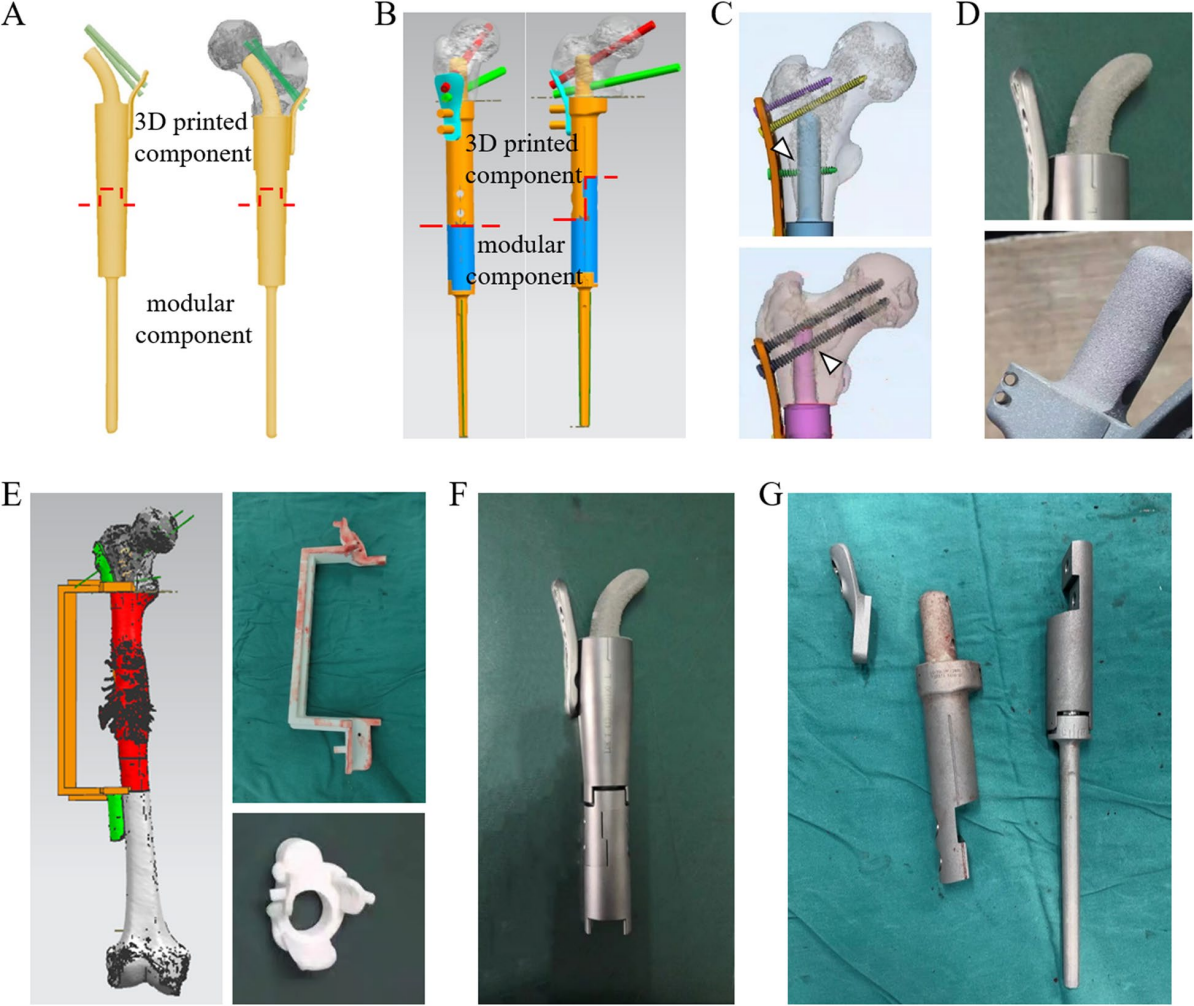
Photographs and design proposals of new-designed 3D-printed prostheses. **A** The gross view of Type A prosthesis on design proposal showing the 3D-printed component (superior part) and LDK modular component (inferior part). **B** The gross view of Type B prosthesis on design proposal showing the 3D-printed component (superior part) and LDK modular component (inferior part). **C** The 3D-printed proximal stems in Type B prosthesis showing anchorage-slot structures (arrowheads) fixing the locking screw inserted into calcar femorale or spongy bone. **D** The finished 3D-printed curved stem and anchorage-slot stem. **E** 3D-printed osteotomy guide plate on design proposal and photograph of finished 3D-printed osteotomy guide plate and reaming guide plate. **F** A finished Type A prosthesis showing the 3D-printed proximal bone-implant interface, distal bone-implant interface, and curved stem. In this prosthesis, the patient-specific curve radius, stem diameter and length were 140°, 15 mm, and 69 mm, respectively. **G** The finished Type B prosthesis showing the 3D-printed proximal bone-implant interface, distal bone-implant interface, and anchorage-slot stem

In this study, two types of 3D-printed customized intercalary prostheses (Type A and Type B) were designed to be implanted in diaphyseal and proximal metaphyseal femurs when massive bone defects caused by tumors appeared (Fig. 2). Both types of prostheses were utilized for the status when the length of proximal bilateral residual cortical bones was insufficient to anchor the usual intramedullary stem. In Type A prostheses, a 3D-printed curved stem in the proximal femur was implemented instead of a traditional “straight” stem with a patient-specific curve radius, stem diameter and length (Fig. 2A). Meanwhile, a 3D-printed customized proximal stem that possessed anchorage-slot structures, which was designed to accommodate the locking cross screw, was fabricated for each Type B prosthesis (Fig. 2B, C). For Type B, the diameter of the proximal stem was determined on the width of the individual medullary cavity to maximize bicortical fixation, and the length of the proximal stem should refer to the level of the osteotomy plane. Meanwhile, the length of the proximal stem must offer sufficient distance for the accommodation of the matched screw (Fig. 2C). After the 3D-printed implants were fabricated, the proximal stems were fitted with titanium or hydroxyapatite (HA) coatings (Fig. 2D), and other post-processing procedures, including thermal disposal, surface polishing, rinsing and irradiating sterilization, were also conducted. The customized stems were fixed with either press-fit or bone cement (polymethylmethacrylate) based on the individual factors of each patient, including oncological prognosis, amount of physical activity, bone conditions, valid press-fit length, response to chemotherapy, age, and patient’s will. The fatigue test was conducted to determine the mechanical safety of the prosthesis for each patient. As previously described, minimum to maximum stresses were applied on the fabricated prostheses with a compressive loading ratio of 0.1 and a frequency of 15 Hz [26]. In this study, the maximum contact force corresponds to a load of 250% of the body-weight of each patient on the proximal stem based on previous standard [27]. The fatigue tests were ended when the stiffness of the testing specimens had increased 10 percents of their initial value. A prosthesis was regarded as mechanical safe if the specimen did not fail after 10^6^ cycles of loading, and the test was stopped [26].

The 3D-printing technique was also applied in the osteotomy guide plates for accurate tumor resection and the bone-implant interfaces for accelerating rigid biological fixation (Fig. 2E). All osteotomy planes were precisely measured and planned on preoperative magnetic resonance imaging (MRI) and CT scanning of the whole thigh.

Reaming of the canal prior to insertion of the proximal stem was performed under the 3D-printed reaming guide plate (Fig. 2E), and the cancellous bone was impacted around the 3D-printed stem to enhance the fixation in the surgery. Each 3D‐printed prosthesis has porous titanium interfaces allowing bone in‐growth with pore sizes of 450 to 600 μm and a porosity of 20%. All the 3D-printed components can cooperate with the modular endoprosthesis system (LDK, LTD, Beijing, China) in our hospital. All implants were designed by the surgeons who participated in this study and fabricated by Thytec Co., Ltd., Shanghai, China (Fig. 2F, G).

### HU *value thresholding*

**Fig. 3:**
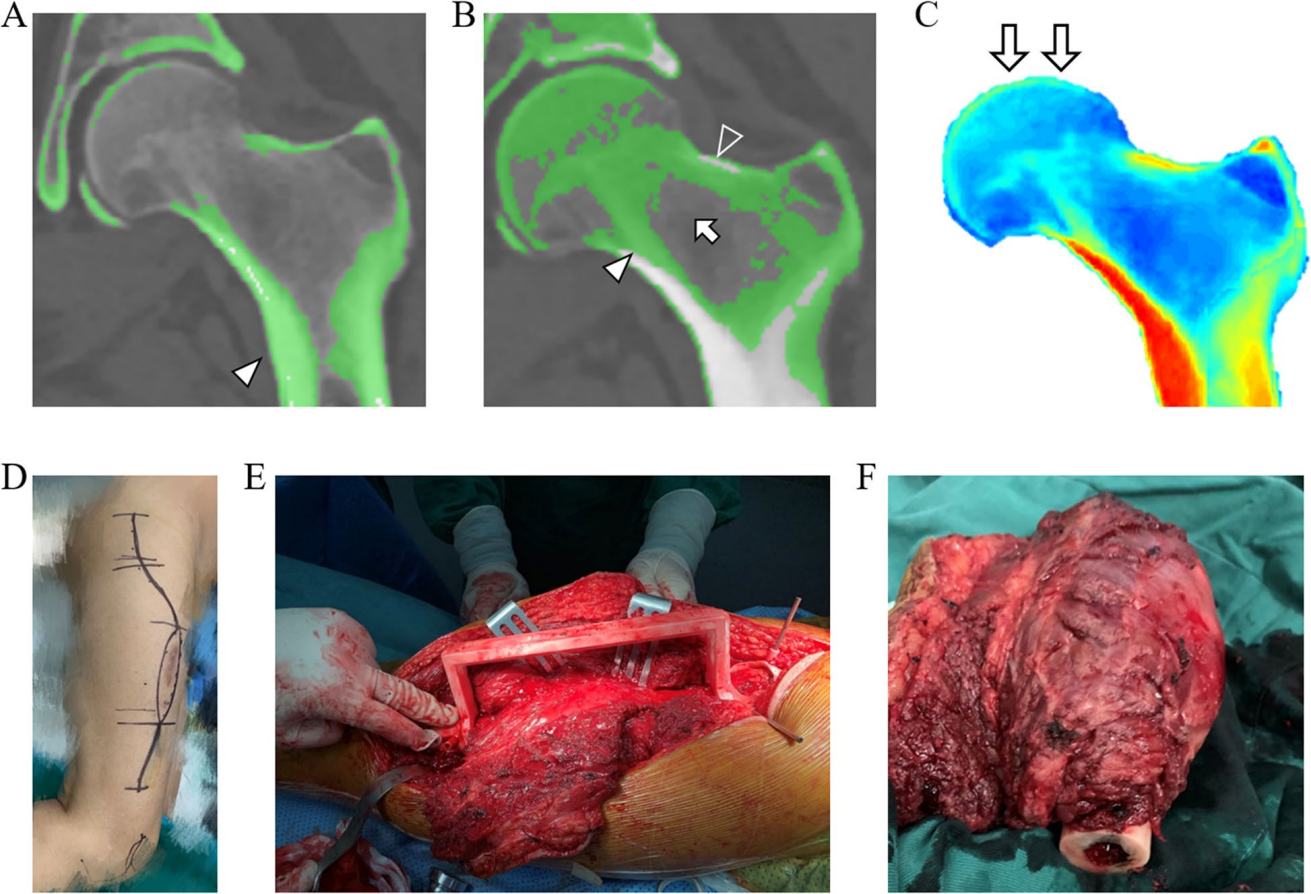
Illustration of HU value thresholding, FEA and intraoperative surgical techniques. **A** The identification of calcar femorale (arrowhead) and lateral cortical bone based on HU value thresholding. **B** The identification of trabecular bone based on HU value thresholding showing principle compressive trabecular bone (arrowhead), principle tensile trabecular bone (empty arrowhead) and Ward triangle (arrow). **C** The biomechanical distribution of the proximal femur analyzed by finite elements showing the direction and location of the applied loading force. **D** The lateral approach for the exposure of the proximal and middle thigh. **E** Intraoperative application of the 3D-printed osteotomy guide plate. **F** The resected tumor-involved segment

We processed the CT scans with the voxel size of 0.70 × 0.70 × 1.00 mm. To scientifically distinguish the structure of calcar femorale and trabecular bone with their peripheral intraosseous region, thresholding technology based on the HU value was applied in this study. Based on previous scientific investigations, the range used for thresholding the trabecular bone region was 100–600 HU, and 600 HU was determined as the CT density of the corticocancellous interface in the proximal femur [28, 29]. Utilizing the image-control software (Mimics), we extracted the HU value in the DICOM data of the CT images from the nine patients to form the corresponding masks, which were sequentially converted to the 3D mesh models [29]. The standard thresholding values were set to determine the regions of calcar femorale and trabecular bone (Fig. 3A, B). To acquire more specific analysis results, manual segmentation may also be applied to better define the margins of cortical and trabecular bone, and relevant verification was conducted by a proficient radiologist. Bone models were generated for cortical and trabecular bone. What should be paid high attention to in the initial design regimen is the adequate anatomic adjacency between the calcar femorale and proximal stem. In addition, the insertion of proximal screws was planned to maximally retain the principle compressive and tensile trabecular bone and calcar femorale.

### Finite element analysis

In this study, the femur was modeled as an elastic and nonhomogenous material by assigning a specific Young’s modulus to each element. A 3D model of the femur was generated with MIMIC software. The body mesh was divided, and the tetrahedron elements were utilized to produce mesh models [30]. To calculate each element’s modulus of elasticity, the HU value was extracted from CT images and was calculated at the central point of each element. CT intensity values were used to assign mechanical properties for cortical and trabecular bone and were calculated by averaging the brightness of pixels within each element. The apparent bone density ρ phantom established a linear relationship with the HU value from CT images. Interior bone density was converted to elastic modulus for each element based on an empirical relationship specific to bone. Equations 1 and 2 were applied to determine the apparent density (ρ, g/cm3) and Young’s modulus (E, MPa) of each element, respectively [30]. The value of Poisson’s ratio was 0.3.

1 $$\rho =0.00069141\times HU+1.026716$$

2 $$E=2017.3{\rho }^{2.46}$$

According to the weight of each patient, the corresponding mimetic force was applied to indicate the stress distribution (Fig. 3C). Referring to the mechanical analysis of finite elements, the proximal stem should be implanted to obey the individual stress distribution and biochemical features to the greatest extent, as should the direction of screws. With the collaborative consideration of the analysis of HU values and finite elements, which were conducted in all patients preoperatively, the surgeons and engineers were able to design the 3D-printed customized prosthesis.

### Surgical techniques

After routine surgical preparation, a lateral approach that may diminish the probability of neurovascular injury was preferentially recommended to dissect the soft tissue and expose the surgical field (Fig. 3D). Tumor resection was conducted under the principles to acquire safe margins based on the preoperative MRIs and CT images (Fig. 3E). Generally, the osteotomy level was determined to be at least 3.0 cm, surpassing the lesion area, which included the edema and reactive zone to achieve a wide margin (Fig. 3F). The personalized 3D‐printed osteotomy guide plate was applied in each patient. The osteotomy was conducted strictly following the preoperative plan after the proximal and distal osteotomy guide plates were riveted in the proper position and therefore allowed the 3D-printed porous interfaces on the implant to fit the residual bone perfectly on the osteotomy planes (Fig. 3E).

The individual 3D-printed stems with titanium/HA coatings were inserted into the proximal femoral metaphysis, maximizing the anatomic reservation and biomechanical distribution. After confirming the locations of all bone-implant interfaces and stems, screws were inserted to enhance primary stability according to the preoperative design. The cannulated or locking screws made of titanium were applied with length of 17–90 mm and diameter of 3.5–7 mm in this study. In the Type A prosthesis, two lateral supplementary tensile screws were placed in the spongy bone region, and locking screws and tensile screws were inserted into the calcar femorale and proximal cancellous bone in the Type B prosthesis. To achieve more reliable and standard screw holes, a drill guide attached to the prosthesis but not a free-hand guide was applied to drill the screw holes in all endoprostheses. The fixation plate was utilized if possible. If the lesser trochanter was partially resected, the insertion of iliopsoas needed to be reconstituted for suturing the tendon of iliopsoas. The operative duration, bleeding volume, and intraoperative adverse reactions were recorded for each patient, and an immediate postoperative X-ray was performed to ensure the placement of implants.

### Postoperative management, assessment and follow-up

The hip motion on bed was started 7–14 days after surgery. Three to five weeks after the operation were usually recommended as the initial time when patients were able to bear partial weight load with the protection of a brace. The weight‐load gently increased to the full weight‐load. All patients participated in the first radiographic follow-up at approximately 3 months after the surgery and every 3 months thereafter. Whether bone ingrowth and osseointegration occurred between residual bone and the 3D-printed porous interface of implants on the osteotomy plane was observed in each radiographic follow-up, as was that on 3D-printed proximal stems. An absence of pain or instability was regarded as a clinical indicator for bone ingrowth [19]. Major complications were categorized as previously described and recorded [31]. We also evaluated functional amelioration by measuring the preoperative and postoperative Harris Hip Score (HHS) and Musculoskeletal Tumor Society (MSTS) score [32, 33]. The cost of the surgeries was also recorded.

### Statistical analysis

Data were analyzed using SPSS 21.0 and MATLAB 2016b. The Kolmogorov- Smirnov (K-S) test was used to analyze the distributed range of each measurement. A paired *t*-test was used to analyze the significant differences between the preoperative and postoperative function scores. Statistical significance was set at *P* < 0.05. *P* < 0.01 was considered highly statistically significant.

## Results

### Radiographic measurement

On reconstructed sagittal and coronal CT images, the proximal end and diaphyseal region in normal mature femurs (*n* = 50) were divided into three ROIs. Based on the radiographic measurement and the analysis by the K-S test, D1 and D2 were 24.28 ± 5.51 mm (95%CI: 13.48–35.08 mm) and 64.85 ± 4.62 mm (95%CI: 55.79–73.91 mm), respectively (Table 2). In the region superior to the inferior edge of the lesser trochanter (ROI-1 and ROI-2), the maximum valid length of bilateral cortical bone that can steadily fix the stem was 10.53 ± 2.82 mm (95%CI: 5.00–16.06 mm). The measurement of the calcar femorale was 38.41 ± 5.06 mm, with an average thickness of 7.24 ± 1.20 mm and density of 809.9 ± 130.32 HU (Table 2).

**Table 2:** Radiographic measurements in 50 normal femurs

Variables	Value
Mean distance of D1 (95%CI) (mm)	24.28 ± 5.51 (13.48–35.08)
Mean distance of D2 (95%CI) (mm)	64.85 ± 4.62 (55.79–73.91)
Maximum valid length of bilateral cortical bone in ROI-1 and ROI-2 (95%CI) (mm)	10.53 ± 2.82 (5.00–16.06)
Calcar femorale (mm)	38.41 ± 5.06 (28.49–48.33)
Thickness of calcar femorale (mm)	7.24 ± 1.20 (4.89–9.59)
Density of calcar femorale (HU)	809.90 ± 130.32 (554.47–1065.33)

*D1* the distance between the trochanteric fossa and the superior edge of the lesser trochanter, *D2* the distance between the trochanteric fossa and the inferior edge of the lesser trochanter, *ROI* region of interest, *HU* Hounsfield Unit. The 95% confidence intervals were calculated by the K-S test

### Individual 3D-printed implant application

After the surgical method and margin were determined, the prosthesis selections of nine patients were also finalized. Type A prostheses were applied in 2 patients, in whom the mean thickness and density of the calcar femorale were 6.4 mm and 649 HU, respectively. Type B prostheses were utilized in the other 7 patients. The proximal osteotomy planes of two Type A patients were located in ROI-2, while the proximal osteotomy level in all Type B patients was determined within either ROI-2 or ROI-3 (Table 3).

**Table 3:** Individual measurements and implant properties for each patient

ID	Calcar femorale (mm)	Thickness of calcar femorale (mm)	Density of calcar femorale (HU)	Region of proximal osteotomy level	Region of distal osteotomy level	Type of prosthesis	Maximum valid length of bilateral cortical bone in ROI-1 and ROI-2 (mm)	Reserved volume of principle compressive trabecular bone	Reserved volume of principle tensile trabecular bone
1	35.4	6.7	688	ROI-2	ROI-3	A	10.7	0.94	0.91
2	29.8	6.1	610	ROI-2	ROI-3	A	8.5	0.91	0.90
3	37.8	9.1	857	ROI-2	ROI-3	B	11.1	0.92	0.93
4	39.6	7.4	831	ROI-3	ROI-3	B	10.2	0.93	0.87
5	42.3	7.2	896	ROI-3	ROI-3	B	9.6	0.98	0.89
6	44.4	8.5	875	ROI-3	ROI-3	B	11.4	0.93	0.85
7	36.1	7.1	785	ROI-3	ROI-3	B	9.6	0.89	0.90
8	39.2	7.3	820	ROI-3	ROI-3	B	9.4	0.91	0.87
9	37.2	8	972	ROI-3	ROI-3	B	10.8	0.92	0.88

*HU* Hounsfield unit

**Fig. 4:**
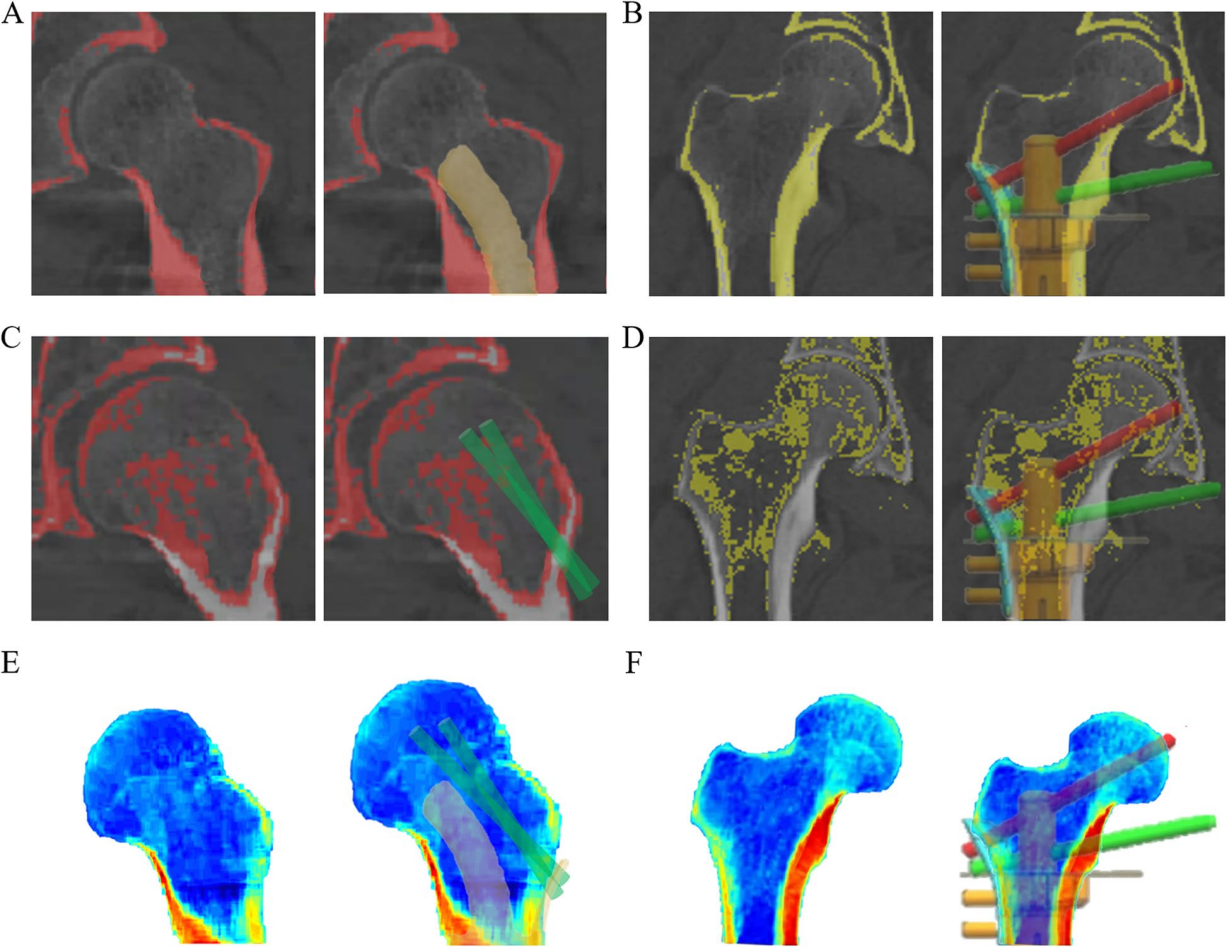
Anatomic determination by HU value and biomechanical analysis by finite element in 3D-printed implants. **A** The preplanned position of the proximal curved stem in the Type A prosthesis, with the optimized preservation and abutment of the calcar femorale (thresholding by HU value). **B** The preplanned position of the proximal anchorage-slot stem in the Type B prosthesis, with the optimized locking screw insertion in the calcar femorale (thresholding by HU value). **C** The preplanned position of proximal screws in Type A prosthesis, with the optimized preservation of trabecular bone (thresholding by HU value). **D** The preplanned position of the proximal anchorage-slot stem and locking screws in Type B prostheses, with the optimized preservation of trabecular bone (thresholding by HU value). **E** The preplanned position of the proximal curved stem and screws in the Type A prosthesis obeying the stress distribution defined by FEA. **F** The preplanned position of the proximal anchorage-slot stem and locking screws in Type B prostheses obeying the stress distribution defined by FEA. Images (**A**, **C**, **E**) from Patient 1. Images (**B**, **D**, **F**) from Patient 3

In the images thresholding by HU values, the principle tensile bone trabecula, principle compressive bone trabecula, and calcar femorale were distinctly presented (Fig. 4A-D). The curved stems in two Type A prostheses and the anchorage-slot stems in seven Type B implants predominantly abutted the calcar femorale to acquire initial stability and subsequently facilitated postoperative bone ingrowth. With the orchestrated screw trajectories, the overall reserved principle tensile and compressive trabecular bone volumes were 86.89 ± 2.42% and 92.56 ± 2.51% in all patients, respectively (Table 3).

Based on the FEA, the stress distribution of each proximal femur was presented. In the region of spongy bone, the screw trajectories were planned obeying the biomechanical distribution and therefore enhanced the ability to transmit the load. A preferential load-carrying was presented upon the medial cortical bone and calcar femorale when compared to the lateral cortical bone in ROI-2, based on which the 3D-printed proximal stem in the Type A endoprosthesis showed a medial tendency with the appropriate curvature, as did the prioritized load-carrying in the Type B prosthesis (Fig. 4E, F).

The proximal and distal individual 3D-printed implant-bone interfaces on the osteotomy plane were successfully manufactured in all implants, as were the corresponding 3D-printed osteotomy guide plates. All the nine finished prostheses did not fail after 10^6^ cycles of loading in the fatigue test.

### Intraoperative procedures

**Fig. 5:**
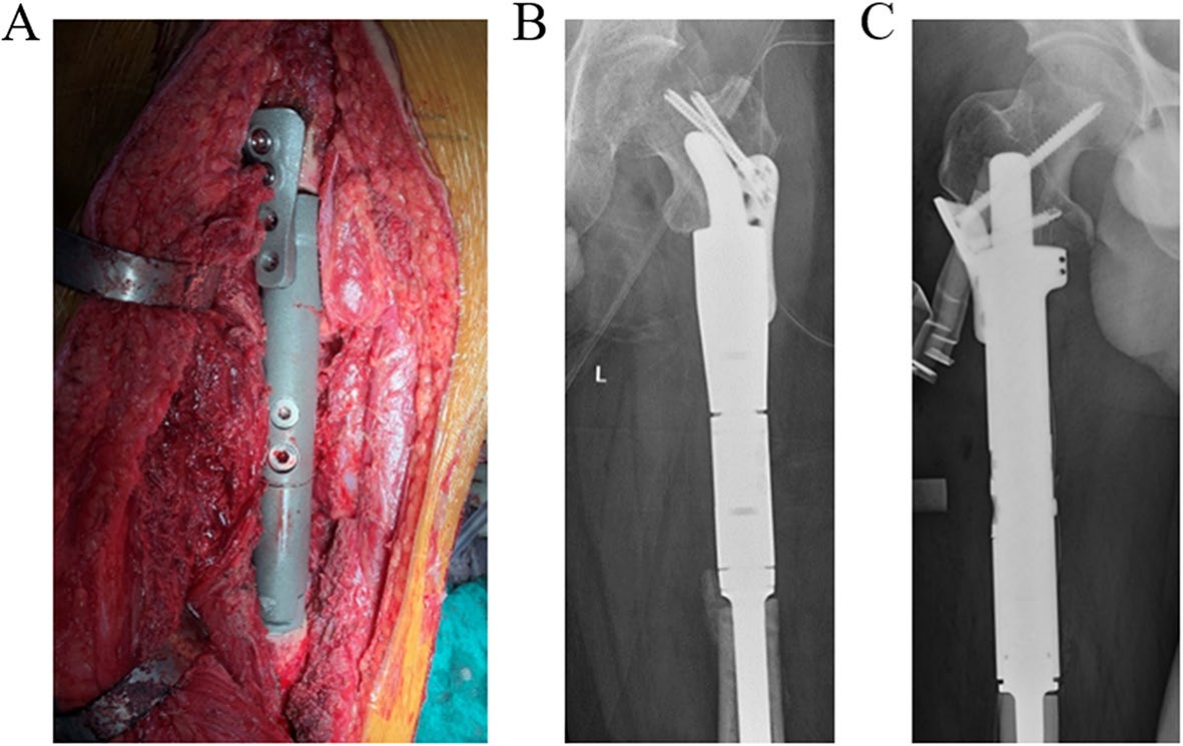
The successful placement of 3D-printed femoral prostheses. **A** The intraoperative implanted 3D-printed prosthesis of Patient 3. **B** The postoperative immediate X-ray of the left femur in Patient 1. **C** The postoperative immediate X-ray of the right femur in Patient 3

All patients underwent the operations uneventfully. The average operative duration and bleeding volume were 325.00 ± 62.60 min and 922.22 ± 222.36 ml, respectively. No intraoperative adverse reactions occurred. The average distance between the trochanteric fossa and the proximal osteotomy plane was 73.00 ± 19.63 mm, while the mean osteotomy length was 182.22 ± 14.45 mm (Table 4). The calcar femorale and trabecular bone were well preserved as planned. Under the 3D-printed osteotomy guide plate and reaming guide plate, the reamed residual bone matched the 3D‐printed interface of the endoprostheses perfectly on all osteotomy planes. The proximal 3D-printed stems of implants were anchored with bone cement and press‐fit manner in 5 and 4 patients, respectively. (Table 4) The 3D-printed Type A or Type B prostheses were successfully implanted in all patients, and lateral fixation plates were implemented in all patients (Fig. 5). We reestablished the insertions of iliopsoas in three patients to acquire a firmer fixation when suturing the muscle tendon. The average cost of the operations was $ 5325.40 ± 376.46.

**Table 4:** Intraoperative data

ID	Osteotomy length (mm)	Distance between proximal osteotomy plane and trochanteric fossa (mm)	Operative duration (minutes)	Bleeding volume (ml)	Adverse reaction	Proximal stem fixation
1	165	51	315	1000	none	press-fit
2	167	45	315	1200	none	press-fit
3	179	52	420	1200	none	press-fit
4	184	74	290	1000	none	bone cement
5	195	75	320	600	none	bone cement
6	190	95	345	1000	none	bone cement
7	183	99	420	900	none	bone cement
8	158	81	270	600	none	bone cement
9	201	85	285	800	none	press-fit

### Postoperative follow-up

**Fig. 6:**
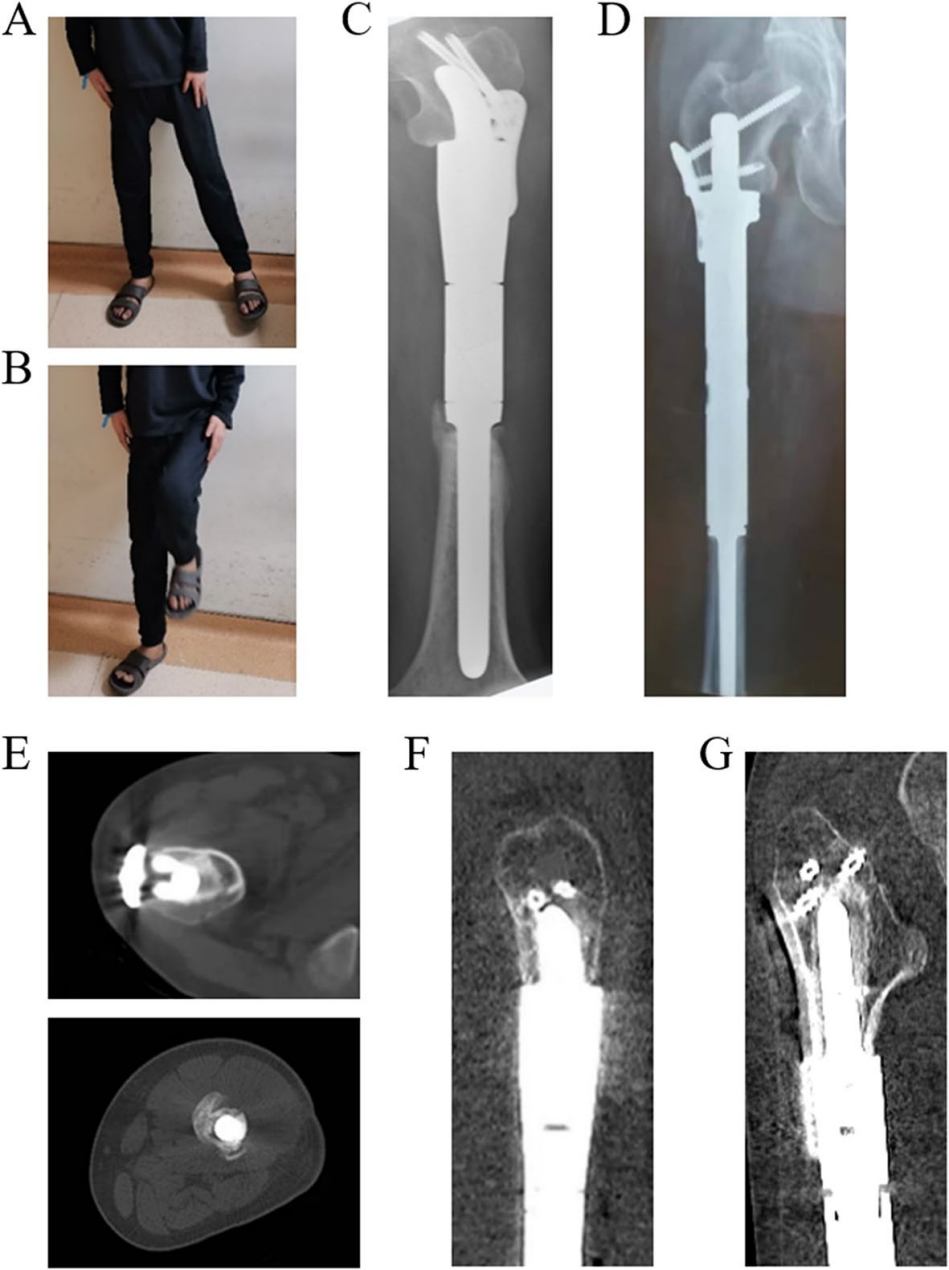
The postoperative functional and radiographic outcomes in the follow-up. **A** and **B** The normal hip range of motion of abduction **A** and flexion **B** in Patient 9 at five months after surgery. **C** X-ray of the left femur at 99 days after surgery of Patient 1 showing reliable biological fixation. **D** X-ray of the right femur at 85 days after surgery of Patient 3 showing reliable biological fixation. **E** Bone ingrowth on the bone-implant interface of the 3D-printed stem and screw (Patient 6) or osteotomy plane (Patient 1) at six months after surgery on axial CT image. **F** Bone ingrowth on the bone-implant interface of the 3D-printed curved stem and screws at six months of Patient 1 after surgery on reconstructed sagittal CT image. **G** Bone ingrowth on the bone-implant interface of the 3D-printed anchorage-slot stem and screws at six months of Patient 6 after surgery on reconstructed coronal CT image

At a mean follow-up of 26.00 ± 10.50 months (range, 10–40 months), five patients were disease free, while oncological events were observed in four patients (Table 5). The rate of implant survival was 100%. Both HHS and MSTS scores showed a significant difference between the preoperative and postoperative evaluations (HHS 69.22 ± 14.64 vs 91.33 ± 2.24, *P* < 0.001, and MSTS 16.89 ± 7.08 vs 27.67 ± 1.73, *P* < 0.001, respectively). The hip range of motion was acceptable, with an average initial partial weight bearing time of 27.56 ± 3.75 days (Fig. 6A, B). No major operative complications occurred in the follow-up, and bone ingrowths on the bone-implant interfaces and proximal 3D-printed stems were observed in all patients with the earliest time of 56 days (Fig. 6C). Rigid biological fixation was confirmed by both radiographic and clinical evidence in all patients (Fig. 6D, E).

**Table 5:** Oncological, clinical, radiographic and functional outcomes in the follow-up period

ID	Follow-up duration (months)	Survival status	Implant survival	Preoperative HHS	Postoperative HHS	Preoperative MSTS	Postoperative MSTS	Major complication	Initial time for partial weight-load (days)	Initial time for bone ingrowth (days)
1	30	NED rl	survival	44	88	5	26	-	23	99
2	33	NED	survival	63	90	16	28	-	29	56
3	36	NED	survival	75	94	20	28	-	32	85
4	40	NED	survival	86	93	22	30	-	28	75
5	10	DFD	survival	83	92	22	28	-	31	82
6	26	NED m	survival	66	91	15	26	-	27	60
7	11	NED	survival	50	88	6	26	-	31	84
8	28	DFD	survival	77	93	23	28	-	26	88
9	20	NED	survival	79	93	23	30	-	21	62
*P* value				*P* < 0.001		*P* < 0.001				

*HHS* Harris Hip Score, *MSTS* Musculoskeletal Tumor Society, *NED* alive with no evidence of disease, *NED rl* no evidence of disease after treatment of a recurrence, *NED m* alive after treatment of metastases, *DFD* died from their disease, Patient 1 underwent the amputation for the local recurrence 26 months after surgery; The lung metastasis was found 20 months after surgery in Patient 6; In both HHS and MSTS scores, a highly statistically significance (*P* < 0.001) was observed between preoperative and postoperative assessments

## Discussion

### Necessities of the new-designed 3D-printed prostheses

With the promotion of oncological prognosis in recent decades, patients who suffer tumorous lesions in the extremities currently warrant a more optimistic postoperative functional prognosis, in which how to manage reconstruction for massive oncological bone defects in the diaphysis has become a hot and challenging topic [3, 34, 35]. After numerous attempts are conducted to solve this problem, modular or customized intercalary prostheses have been considered a mainstay to reconstitute diaphyseal massive bone defects in current consensus [9, 36]. However, the issues include insufficient articular mobility, prosthesis-related adverse events, revision surgery, etc. often emerge if the lesion also invades the metaphysis due to the limited length of bilateral cortical bone that is qualified to stabilize the intramedullary stem [7, 8]. The 3D-printed technique has been preliminarily applied for intercalary prostheses to reconstruct the diaphyseal and metaphyseal regions in joint-salvage resections in the tibia, achieving an acceptable level of biomechanical, radiographic and functional prognosis [18]. However, customized oncological intercalary prostheses that reconstitute both the proximal end and diaphysis of the femur with the preservation of the hip joint have rarely been discussed in previous studies [8].

In this study, two types of original 3D-printed intercalary prostheses were described, with their characteristics stated below: (1) The two types of 3D-printed oncological prostheses are suitable and feasible for application in the reconstruction of femoral diaphyseal massive bone defects with limited distances between the proximal osteotomy plane and trochanteric fossa (Fig. 2) (Table 4). (2) The overall assessment of these two types of new-designed prostheses, including intraoperative data, clinical prognosis, radiographic observation and functional amelioration, was satisfactory (Table 5). (3) A comprehensive application of several techniques, including ROI division, radiographic measurement, HU value thresholding, and FEA, was conducted to guide the design and manufacture of these two types of 3D-printed implants (Fig. 4). As far as we know, Type A and Type B prostheses have never been reported in previous studies, nor have their features and postoperative evaluation been elaborated.

### Advantages of the new-designed 3D-printed prostheses

Benefiting from 3D-printed technology, Type A and B implants are successfully designed to solve a critical problem that a traditional stem is unreliable to fix without specific modification in ROI-1 and ROI-2 because the bilateral residual cortical bones are commonly less than 5 cm in these regions (Table 2). Based on the radiographic measurements, a sufficient length but insufficient bilateral cortical bone were presented in ROI-2 for anchoring the traditional stem, implying the possibility of implementing special customized stems with the osteotomy level in this region. In contrast, it seems impracticable to conduct hip-salvage surgery with the utilization of such 3D-printed stems when the osteotomy plane is located within ROI-1 due to the extremely short length of D1. The lesser trochanter has been extensively applied to guide regional division in anatomic and orthopedic research due to its high identifiability [23, 37]; however, the reference of this anatomic landmark is relevantly limited in musculoskeletal oncology. The identification of ROI-1 to ROI-3 facilitated us in making a rapid and direct judgment of whether selecting a Type A or B prosthesis for the reconstruction is appropriate in each patient.

The curved stem in the Type A prosthesis was placed to abut but not to damage the calcar femorale, and the 3D-printed anchorage-slot stem in Type B enabled the precise insertion of a locking screw. In this study, the calcar femorale enabled reliable fixation for proximal stems and screws, especially when the medial cortical bone was limited. Based on the orchestrated screw trajectories, the tensile bone trabecula and compressive bone trabecula, which are the pivotal structures for load transfer and load-carrying in the proximal femoral metaphysis [1], were maximally reserved and therefore maintained their biomechanical support (Fig. 4A-D). According to the biomechanical loading fatigue test, the mechanical safety of both Type A and Type B prostheses was confirmed. Based on previous investigations involving FEA, the selection of a curved stem in the proximal femur was proven to diminish the load-carrying burden of the calcar femorale and lateral cortical bone, and the appropriate application of screws between the implant and femoral head and neck was conducive to the transmission of stress in the proximal femur [38, 39]. An inverted triangle configuration has been well proven for its superiority in biomechanical performance when cannulated screws are applied for femoral neck fracture [40]. In this investigation, the proximal screws were inserted into the proximal spongy region and calcar femorale, and the preserved femoral calcar was also placed in a triangle configuration, which may optimize the load transfer and carrying. Generally, whether to choose a Type A or Type B prosthesis was mainly based on the following factors: the level of the proximal osteotomy plane, the quality of the calcar femorale and the individual characteristics of each patient. To the best of our knowledge, this is the first clinical attempt to apply such 3D-printed intercalary prostheses, which were designed with the integrative consideration of ROI division, radiographic thresholding and biomechanical FEA, to solve the problem that the stem of traditional femoral intercalary prostheses was unable to be fixed assuredly. Moreover, with the application of Type A and B endoprostheses, some joint-sacrificed surgery techniques, such as hip hemiarthroplasty [41], which may lead to relatively imperfect postoperative function, may be altered, especially for lesions that involve the proximal metaphysis but not the epiphysis.

With the help of the corresponding 3D-printed guide plates, the 3D-printed interfaces on the implant fit the residual bones perfectly in this study to avoid prosthetic micromotion in the early stage by promoting osseointegration and diminishing the ingrowth of fibrous tissue. Furthermore, the accurate osteotomy on the proximal plane ensured the precise implantation of personalized prostheses; therefore, minimum deviation would occur in the area where the proximal stem obeyed the anatomical characteristics and biomechanical distribution was placed. Unlike conventional one-time reaming, the maximum diameter for reaming was gradually obtained in this research. With the help of a 3D-printed reaming guide plate, we applied a customized reamer or an equivalent prosthesis model instead of traditional reaming instruments to ream the canal for the proximal stem, and the cancellous bone was tamped at the vicinity of the 3D-printed stem, alternating between reaming and cancellous bone tamping. Such alternation contributes to ensuring the bone graft volume, elasticity, and implant fit. In addition, the personalized shape and porous structure on the bone-implant interface conducted appropriate mechanical induction to accelerate bone ingrowth [18]. Meanwhile, the application of the coatings made of titanium or HA not only enhanced the structural connection between the implant and residual bone but also provided biological stimulation to induce bone ingrowth and osseointegration and enclosed the surface of the stem by dense molecular constructions to prevent the transgression of harmful particles, which may negatively alter bone ingrowth [20] (Fig. 6C-E). In this study, the internal fixation plate was utilized lateral to the femur in all patients, which is beneficial to protecting medial neurovascular structures, providing an adequate surgical field, and facilitating its placement. In addition to strengthening the stability of the endoprosthesis, the internal fixation plate, which possessed the corresponding holes for orientation in the Type B prosthesis, can further increase the accuracy of inserting the locking screws, which have been confined in the 3D-printed anchorage-slot, as another confirmation. We summarized the specific characteristics of the two types of new-designed implants (Table 6) because their primary application achieved uneventful operative progress and acceptable postoperative outcomes in this study.

**Table 6:** The characteristics of two new-designed 3D-printed intercalary prostheses

Type	A	B
Reconstructed regions	ROI 2–3	ROI 2–3 or ROI-3
Regions of proximal stem and screws	ROI 1–2	ROI 1–2
Proximal osteotomy plane	ROI 2	ROI 2 or ROI 3
Distal osteotomy plane	ROI 3	ROI 3
Proximal 3D-printed stem	Curved stem	Anchorage-slot stem
Proximal screws	Tensile screws to enhance the stability and load-transfer	Cooperative utilization of locking screw(s) and tensile screws to provide reliable initial mechanical stability, enhance load-transfer and facilitate the later biological fixation
Proximal screw insertions	Spongy bone	Calcar femorale and spongy bone
Osteotomy implementation	Under 3D-printed osteotomy guide plate	Under 3D-printed osteotomy guide plate
3D-printed bone-implant interfaces	Both proximal and distal interfaces	Both proximal and distal interfaces
Anatomical advantages	Maximum retainment of calcar femorale and load-bear bone trabecula	Maximum retainment of calcar femorale and load-bear bone trabecula
Biomechanical advantages	Obey the biomechanical distribution Provide strong supporting for the load-carrying of calcar femorale and medial cortical bone Enhance the load-transfer from the femoral head and neck	Obey the biomechanical distribution Stabilize the mechanical system of proximal metaphysis and calcar femorale Enhance the load-transfer from the femoral head and neck
Fixation plate	Lateral plate is preferential	Lateral plate is preferential
Reestablishment of attachment	Iliopsoas insertion	Iliopsoas insertion if resected

Reconstructing massive or even “ultra-critical sized” bone defects has been verified as a remarkable advantage of 3D printing technology, especially for reconstruction when insufficient residual cortical bones that are unable to immobilize the stems exist [18, 19]. Some scholars have described the successful application of 3D-printed intercalary prostheses in tibial “ultra-critical sized bone defects” [18], while Liu et al. demonstrated that personalized intercalary prostheses based on 3D printing techniques may facilitate knee joint‐preserving tumor resection if the massive bone defect involves the distal femur or proximal tibia [19]. The views stated above were supportive of the reasonability and feasibility of Type A and B prostheses. In this study, massive bone defects, which are generally considered bone defects, were > 15.0 cm in all patients [18]; however, with precise ostectomy and perfectly matched interfaces, a shortened operative duration, acceptable intraoperative bleeding volume, reliable osseointegration, optimistic functional outcomes and low incidence of complications were acquired (Tables 4 and 5). Therefore, the Type A and B intercalary prostheses that were elaborated in this study proved several universal advantages that have been certified in the 3D-printed intercalary prostheses that were utilized for the diaphyseal bone defect of other long bones according to previous studies [18–20]. Moreover, based on previous studies, the cost-effective evaluation of endoprostheses was favored for osteoarticular allografts when utilized for massive bone defects [42]. Thus, this reconstruction method can be regarded as a cost-effective choice due to its satisfactory incremental cost-effectiveness ratios and low postoperative revision rate and major complication rate [42].

## Optimistic overall prognosis

Based on the improvement in prosthetic material and modification in surgical technique, acceptable functional outcomes have been reported for applying conventional intercalary prostheses for the reconstruction of a single diaphyseal region [40]. However, a relatively negative functional prognosis may be presented when traditional customized or modular endoprostheses are utilized in the femur if the proximal residual cortical bones are insufficient compared to the assessment of the new-designed 3D-printed implants in this study [43]. Multiple factors may contribute to the optimistic motor function achieved in these two novel prostheses. First, it has been extensively verified that the appropriate design of intercalary prostheses, which enables the stems to possess adequate length and diameter, can benefit the functional prognosis in previous studies [3, 7]. Thereby, we designed the curved stem and anchorage-slot stem, which both provided sufficient length and surface to take into account both the immediate mechanical support and the facilitation of peripheral bone ingrowth. As a consequence, these implants acquired both early and late postoperative stabilization (Fig. 6). Second, applying bone cement and press‐fit, which are two common techniques to fix stems, tends to provide early and late stabilization, respectively [36]. Therefore, how to scientifically select the fixation method may be critical to affect implant survival and implant-related adverse events. In this study, whether the 3D-printed stem was fixed by bone cement or press-fit was determined upon the individual characteristics of each patient after comprehensive consideration rather than an invariable general standard. Based on multiple criteria, neither shifting nor loosening of the stem was observed in this study. Third, the locking screws were fixed firmly and precisely based on 3D-printed anchorage-slot stems and therefore provided strong supplementation to enhance the initial prosthetic steady before reliable osseointegration formed. Fourth, the insertion of iliopsoas was reconstituted in the patients who underwent resection of the partial lesser trochanter, and an ameliorated HHS score (69.22 ± 14.64 vs 91.33 ± 2.24, *P* < 0.001) was obtained in those patients. Last, starting postoperative exercises too early may increase the risks of implant-related adverse events; however, poor postoperative function of the joint or limb may emerge if these exercises are begun too late [43, 44]. Therefore, it is important to determine an appropriate time for the initial free and load-bearing training. In this study, no functional complications occurred following the initial hip motions on bed or partial weight-load activities. The radiographic prognosis was also satisfactory in this investigation, as evident bone ingrowth and osseointegration were observed on postoperative X-ray and CT images (Fig. 6C-G). Clinically, no major complications are presented in the follow-up, supporting the superiority of the overall prognosis of these new-designed 3D-printed femoral intercalary prostheses.

## Limitations of this study

The main limitations in our study are stated as follows. First, it must be acknowledged that the number of patients enrolled in this study was limited with a relatively short follow-up period. Because the original design concept of such a novel 3D-printed intercalary has just been implemented in recent years, the universality of our results would be better confirmed if multicenter, larger‐scale, and long-term investigations were conducted in the future. Second, the fabrication of such 3D-printed prostheses requires a relatively long period and a specialized team that contains both senior surgeons and experienced engineers. Third, traditional medical CT but not industrial CT was performed in this research to guarantee clinical safety; therefore, artifacts between the implant and residual bone were inevitably generated. We applied a biomechanical loading test instead of the postoperative FEA to decrease information bias; however, such technology may show a paucity of verification of optimal load transfer on bone-implant assembly and identification of stress shielding at the bone-implant interface. Last, the design and manufacture of these special implants integrate multiple techniques, such as radiographic measurement, HU value thresholding, and FEA, and therefore require multidisciplinary clinical researchers.

## Conclusion

Two new-designed 3D-printed femoral intercalary prostheses, which have acquired acceptable clinical, radiographic and functional prognoses in the primary application, were described in this study, and their specific characteristics were elaborated. Based on the implementation of such 3D-printed implants, patients who require reconstruction for massive femoral diaphyseal defects with limited proximal residual bone may benefit from functional amelioration and salvage of the hip joint. Several scientific methodologies were cooperatively utilized to optimize the design scheme of these 3D-printed femoral intercalary endoprostheses in this investigation, which may provide novel alternatives to improve the design proposals of 3D-printed implants in the future.

## Data Availability

The data presented in this study are available on reasonable request from the corresponding author.

## Abbreviations

ROI: Region of Interest
HU: Hounsfield Unit
FEA: Finite Element Analysis
CT: Computed Tomography
HA: Hydroxyapatite
MRI: Magnetic Resonance Imaging
HHS: Harris Hip Score
MSTS: Musculoskeletal Tumor Society
